# Research on Fatigued-Driving Detection Method by Integrating Lightweight YOLOv5s and Facial 3D Keypoints

**DOI:** 10.3390/s23198267

**Published:** 2023-10-06

**Authors:** Xiansheng Ran, Shuai He, Rui Li

**Affiliations:** School of Mechatronics and Vehicle Engineering, Chongqing Jiaotong University, Chongqing 400074, China; ranxiansheng@cqjtu.edu.cn (X.R.); 622210990044@mails.cqjtu.edu.cn (R.L.)

**Keywords:** YOLOv5s lightweight, maxpool cross-scale feature aggregation, context information fusion, 3D keypoints, eye and mouth features, fatigued-driving detection

## Abstract

In response to the problem of high computational and parameter requirements of fatigued-driving detection models, as well as weak facial-feature keypoint extraction capability, this paper proposes a lightweight and real-time fatigued-driving detection model based on an improved YOLOv5s and Attention Mesh 3D keypoint extraction method. The main strategies are as follows: (1) Using Shufflenetv2_BD to reconstruct the Backbone network to reduce parameter complexity and computational load. (2) Introducing and improving the fusion method of the Cross-scale Aggregation Module (CAM) between the Backbone and Neck networks to reduce information loss in shallow features of closed-eyes and closed-mouth categories. (3) Building a lightweight Context Information Fusion Module by combining the Efficient Multi-Scale Module (EAM) and Depthwise Over-Parameterized Convolution (DoConv) to enhance the Neck network’s ability to extract facial features. (4) Redefining the loss function using Wise-IoU (WIoU) to accelerate model convergence. Finally, the fatigued-driving detection model is constructed by combining the classification detection results with the thresholds of continuous closed-eye frames, continuous yawning frames, and PERCLOS (Percentage of Eyelid Closure over the Pupil over Time) of eyes and mouth. Under the premise that the number of parameters and the size of the baseline model are reduced by 58% and 56.3%, respectively, and the floating point computation is only 5.9 GFLOPs, the average accuracy of the baseline model is increased by 1%, and the Fatigued-recognition rate is 96.3%, which proves that the proposed algorithm can achieve accurate and stable real-time detection while lightweight. It provides strong support for the lightweight deployment of vehicle terminals.

## 1. Introduction

With the rapid development of industrial technology, there have been fundamental changes in the structure of transportation. Although the popularity of cars has made travel more efficient and convenient, it has also brought about inevitable traffic accidents. According to data statistics, the main causes of traffic accidents are closely related to fatigue, drunk driving, overload, and speeding. In particular, fatigued driving accounts for 14–20% of all traffic accidents, with the occurrence rate of major traffic accidents reaching as high as 43%. Traffic accidents caused by large trucks and on highways account for approximately 37% [1]. This is because after long periods of intense driving, the muscles and mental state of drivers become relaxed and fatigued, leading to a decrease in reaction and anticipation abilities, thereby posing a serious threat to life and the surroundings [2]. Therefore, in-depth research on fatigued-driving detection is of great significance in reducing the occurrence rate of traffic accidents and ensuring personal and property safety.

Currently, research on driver-fatigue detection mainly focuses on the field of road traffic and can be divided into three methods: detection based on vehicle driving characteristics [3,4,5], detection based on driver physiological characteristics [6,7,8], and detection based on computer vision of driver facial features [9,10,11,12,13,14,15,16,17,18,19,20,21,22]. Among them, visual-based detection uses cameras or other image sensors to capture the facial-feature changes or head-movement information of the driver. It uses deep-learning algorithms to locate and analyze eye features (blink frequency, eye aspect ratio, cumulative closed-eye time, etc.), mouth features (degree of mouth opening, mouth aspect ratio, etc.), head-pose features (yaw angle, pitch angle, etc.), and facial-expression features, thus achieving fatigue detection through single or multiple feature fusion. This method has the advantage of non-invasiveness, not only accurately determining the driver’s fatigue level but also issuing timely and effective warnings, making it the main research focus currently. Among the many extractable driver facial features, determining whether fatigued driving is present is particularly important. Therefore, in 1998, an important evaluation index called “PERCLOS [9]” emerged, which can effectively determine fatigued driving based on the percentage of closed-eye time within a specific time period and has been widely used in fatigue identification. In addition, the research by Dziuda et al. [10] further validated the importance of PERCLOS in fatigued-driving detection. They evaluated eight professional truck drivers by calculating PERCLOS, duration of closed eyes, and blink frequency in facial images of the drivers. The research results showed that PERCLOS is a variable important predictive factor and is considered an important determining indicator in fatigued-driving detection research.

Early fatigued-driving detection methods mainly focused on extracting single facial features. Alioua et al. [11] proposed a yawning detection algorithm that used an SVM detector for feature extraction. The accuracy of extracting the mouth region through Hough transformation can reach 98%. Zhang et al. [12] fused long short-term memory networks and convolutional neural networks to propose a fatigued-driving detection method that analyzes time features with an accuracy rate of over 87% for continuous yawning states of drivers. Knapik et al. [13] introduced a more innovative approach by using an infrared thermography model to detect drivers’ yawning states and integrating it into an advanced driver-assistance system, enabling fatigued-driving detection and recognition without interference under both day and night conditions. However, these methods did not consider the issues of feature loss and increased false detection rates caused by occlusion and significant changes in driver posture, and they exhibited poor stability. To address these issues, researchers widely favor the method of facial multi-feature fusion to overcome the drawbacks of single-feature extraction and the interference of the external environment on the driver’s state. Among them, the combination of multi-keypoint localization of MTCNN [14] and Dlib [15] with multiple features such as eyes, mouth, and head pose has been most applied. Deng et al. [16] used the improved MedianFlow face tracking and detection algorithm with MTCNN to locate and track the eyes and fused the information of blink frequency and head position changes to realize fatigued-driving detection. Liu et al. [17] used the multi-task cascaded convolutional neural network MTCNN to locate the five key facial positions of the driver’s eyes and mouth. Based on the PERCLOS criterion and fuzzy inference principle, the eye and mouth fatigue feature parameters were fused to determine the driver’s fatigue level. Liu et al. [18] used the multi-scale block local binary patterns (MB-LBP) and Adaboost classifier to extract 24 facial keypoints. Based on the states of the eyes and mouth, PERCLOS and yawning frequency were calculated, and a fuzzy inference system was used to infer the driver’s fatigue state. However, the five-point localization of MTCNN and 24 facial keypoints cannot fully cover the facial feature region, and the stability and accuracy of feature extraction are easily affected by the external environment. Therefore, some scholars used the Dlib to extract 68 key facial points, which covers more comprehensive facial features, and based on this, they calculated the eye-mouth aspect ratio and combined it with PERCLOS and the driver’s head-pose changes for recognition and judgment. Experimental results showed that the head-pose-estimation method based on the 68 key facial points can accurately determine a fatigue state and has high robustness [19], but it lacks consideration for lightweight processing. In addition, based on Dlib, Li et al. [20] used an improved lightweight network YOLOv3-tiny to build driver identification and fatigue evaluation models through online evaluation. They calculated the driver’s eye-closed time, blink frequency, and yawning frequency, achieving a fatigued-driving detection accuracy of 95.1%, with remarkable effects. However, the weight model of YOLOv3-tiny is still not lightweight enough and needs further optimization for lightweight deployment on in-vehicle terminals. Furthermore, Babu et al. [21] have developed a drowsiness recognition system using Python and Dlib, which includes face detection and head-pose detection. It achieved a 94.51% accuracy in real-time video detection. Cai et al. [22] used multi-thread optimized Dlib to narrow the face-feature region to the real-time changes of the eyes, mouth, and head and fused multiple feature subsets to realize the fatigued-driving signal detection method based on D-S evidence theory. However, a fatal problem that has been largely overlooked in the above studies is the tendency of the Dlib 2D facial landmark extraction library to lose feature points and have poor real-time performance when there are significant changes in the driver’s head pose.

In summary, to achieve effective detection of fatigued driving, high-precision detection of facial features is a primary requirement. However, in most previous studies, in order to improve the detection accuracy of the network model, the limitation of application terminal computing resources was often overlooked. Moreover, traditional methods, such as MTCNN five-point localization and Dlib two-dimensional keypoint extraction, still need improvement in terms of stability and detection speed, and their real-time performance is relatively poor, which to some extent restricts the deployment of fatigued-driving detection systems in onboard terminals. Therefore, the current research focus is on how to improve the accuracy of multi-feature detection of facial features while keeping the model lightweight, and how to efficiently and stably extract facial-feature points to construct a fatigued-driving detection model. To address these issues, firstly, this paper applies lightweight processing of the backbone network using ShuffleNetv2_BD on the basis of the YOLOv5s baseline model. Then, the maxpool cross-scale aggregation module (M-CFAM) and context information fusion module (L-CIFM) are used to promote the fusion of deep and shallow features, enhance the ability of deep features to extract facial information, and reduce information loss in shallow-feature categories. In addition, the CIoU in the baseline model is replaced with WIoU, and the lost functions are reconstructed by using the static focusing mechanism (MF) to accelerate the convergence speed of the model. Finally, based on lightweight facial-feature detection, the Attention Mesh is used to extract 468 three-dimensional facial keypoints and calculate the aspect ratio of the eyes and mouth. A fatigued-driving detection model is constructed based on the fusion of features including the number of continuous closed-eye frames, the number of continuous yawning frames, and the thresholds of eye and mouth PERCLOS. Experimental results show that the model designed in this paper can achieve high-precision real-time detection and judgment with significantly reduced parameter and computational complexity, laying a theoretical foundation for deployment on mobile terminals.

The remaining parts of this article are organized as follows: Section 2 introduces the basic architecture of YOLOv5s and proposes optimization and improvement solutions for face-detection networks. Section 3 extracts fatigue-feature points and constructs a fatigue-determination model. Section 4 validates and discusses the effectiveness of the improved face-detection algorithm and fatigued-driving determination model through experiments. Section 5 presents the conclusion and summarizes the entire article.

## 2. Design of Face-Feature Detection Network

### 2.1. The Basic Architecture of YOLOv5s

**Figure 1:**
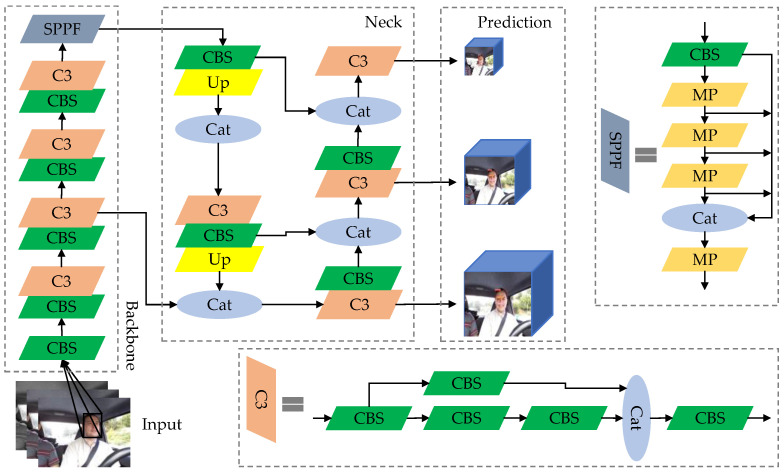
YOLOv5s structure.

As a one-stage object-detection algorithm, YOLOv5 can be divided into five models with progressively increasing scales based on different depth factors and width factors: YOLOv5n, YOLOv5s, YOLOv5m, YOLOv5l, and YOLOv5x. Considering practical application scenarios and computational costs, this article takes YOLOv5s as the baseline model, which consists of the following four components (as shown in Figure 1):

(1) Input: Adopting Mosaic data augmentation, adaptive image scaling, and anchor box computation to enhance model training speed and reduce redundant information.
(2) Backbone Network: By introducing the CBS convolutional structure and C3 module, the backbone network can perform targeted downsampling, selectively preserving detailed information of the target features, and effectively preventing degradation of network performance.
(3) Feature Fusion Network (Neck): The FPN [23] and PAN [24] structures can enhance the network’s feature-fusion capability, reduce information loss during downsampling, and achieve effective fusion of information at different scales, enriching the texture information of shallow features and the semantic structure of deep features.
(4) Prediction: The CIoU [25] loss function is used, which considers the area overlap, aspect ratio, and center point distance between the ground truth box and the predicted box. This ensures a good fit for width and height even when the center points of the ground truth and predicted boxes overlap or are very close. The predicted redundant information is then filtered using NMS (non-maximum suppression) to enhance the effective detection of the target region.

### 2.2. Face Feature Extraction Network of Improved YOLOv5s

#### 2.2.1. Feature Extraction Backbone

The backbone of YOLOv5s utilizes CSPDarknet, which contains multiple deep convolutions for feature extraction, resulting in a relatively high computational load. Therefore, in order to effectively balance the relationship between detection speed and accuracy, and reduce the model’s parameter and computational load, Shufflev2 [26] is introduced as the backbone of the baseline model. It is mainly designed based on ShuffleNet [27] and consists of two parts: the basic unit and the downsampling unit. First, the basic unit adopts Channel Split to split the input feature channels into two paths. One path performs identity mapping to preserve the original features, while the other path utilizes two 1 × 1 standard convolutions and one 3 × 3 depth convolution for dimension reduction and speedup, balancing the channels. Then, Channel shuffle is used to increase information transfer between the two branches and promote feature fusion. The downsampling unit removes the Channel Split and introduces a depth convolution with a stride S of 2 in both channel paths to achieve a lightweight network.

**Figure 2:**
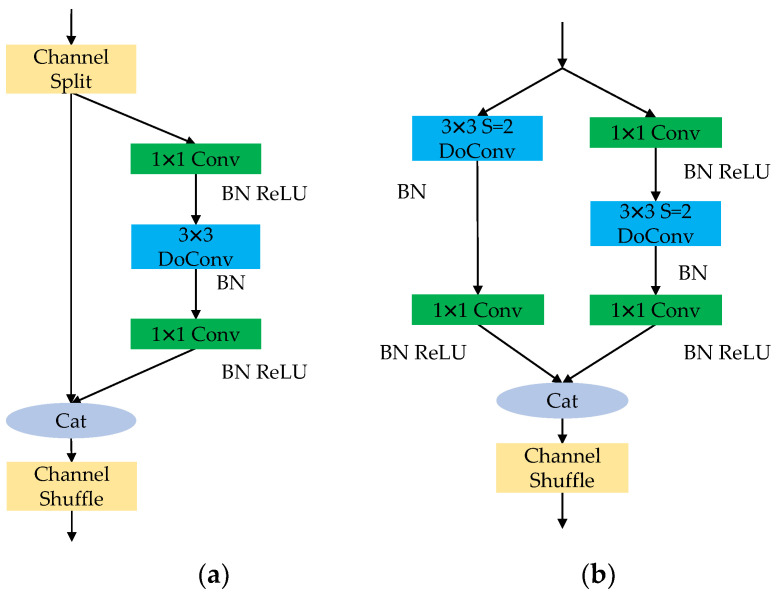
Shuffle_Block structure after depthwise over-parameterized optimization: (**a**) Shuffle_BD 1; (**b**) Shuffle_BD 2.

The adoption of depthwise convolution can effectively reduce the computational and parameter complexity of the model. However, compared to standard convolution, it reduces the search space of convolutional kernel parameters, resulting in a decrease in network representation capacity during feature extraction and fusion. Therefore, the depthwise over-parameterized convolution (DoConv) [28] is introduced to replace DWConv in the Shuffle_Block, allowing the depthwise convolution to be folded into a compact single-layer representation, only one layer is used during inference. The basic unit structure of the improved Shufflenetv2_BD is shown in Figure 2a, and the downsampling unit structure is shown in Figure 2b.

#### 2.2.2. Maxpool Cross-Scale Feature Aggregation

During the driving process, extreme weather or environmental changes may cause confusion or loss of information for shallow facial features (such as closed eyes or closed mouth). Therefore, this paper introduces the Cross-scale Aggregation Module (CAM) [29] between the Backbone and Neck networks and improves it to enhance the fusion of information between different feature levels in the facial region, reducing the loss of shallow category features.

**Figure 3:**
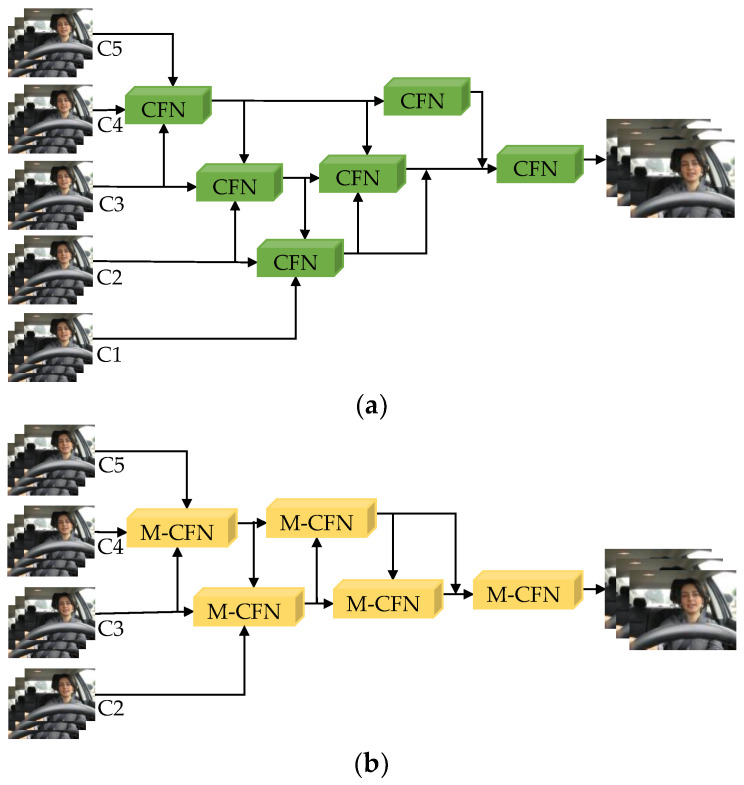
Cross-scale aggregation module before and after improvement: (**a**) CAM; (**b**) M-CFAM.

The CAM structure, as shown in Figure 3a, consists of 5 cross-scale fusion nodes (CFN) arranged in a “V” shape module layout. The intermediate layer of the CFN input is the output of the previous CFN. It integrates the facial features from the backbone network in a bottom-up manner while allowing interaction between the top and bottom layers. The 5-level features of the backbone are aggregated into low, medium, and high-level feature maps, and then the shallow feature expression of the Neck network is enhanced using the 3-level features from different mappings, strengthening the filtering of invalid information.

**Figure 4:**
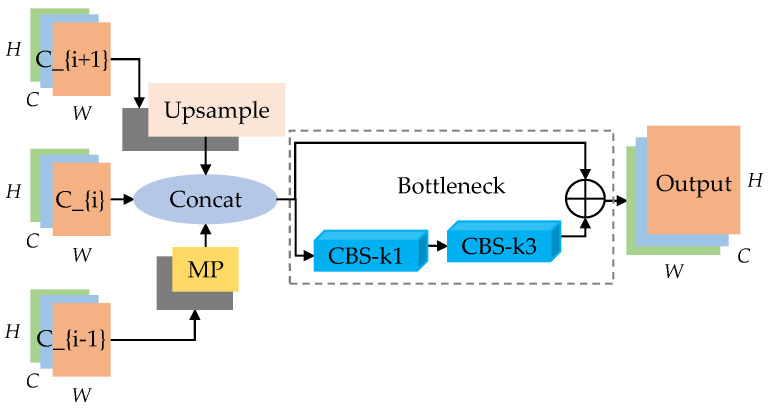
M-CFN.

The improved CAM is called M-CFAM (maxpool cross-scale feature aggregation module), as shown in Figure 3b. In general, it uses the maxpool cross-scale fusion node M-CFN to aggregate the three adjacent features C_{i − 1}, C_{i}, and C_{i + 1} (2 ≤ i ≤ 4) of the backbone as inputs to the continuous nodes for fusion with the C1, C2, C3, and C4 features of the backbone. The structure of M-CFN is shown in Figure 4. Firstly, it combines standard convolution and residual connections to reconstruct the bottleneck structure, reducing the loss of feature information caused by multi-layer continuous convolutions and reducing computational complexity. Secondly, considering that the initial module downsampling Focus enhances the connection between facial features, it increases the difficulty of training in deep convolutions. Moreover, frequent slicing operations are not friendly to embedded platforms, and network quantization operations do not support the Focus module. In this paper, the Focus slicing operation is replaced by a maxpool layer (MP) with a stride of 2 to fully preserve the upper facial texture features and further reduce computational complexity. This forms the “trapezoidal” maxpool cross-scale feature aggregation module M-CFAM.

#### 2.2.3. Lightweight Contextual Information Fusion Module

Due to its close relationship with the surrounding area information, facial features play a crucial role in feature extraction and face detection. Therefore, in order to enhance the fusion of contextual information in Neck’s C3 and improve the feature-extraction capability of categories, this paper introduces the idea of the RFB [30] module and constructs a lightweight contextual-information-fusion module (L-CIFM) based on DoConv and the efficient multi-scale attention module (EAM) [31] to replace C3 in the Neck network. This reduces the equivalent computation with traditional convolutional layers, improves the training speed of deep linear networks, and optimizes overall performance.

**Figure 5:**
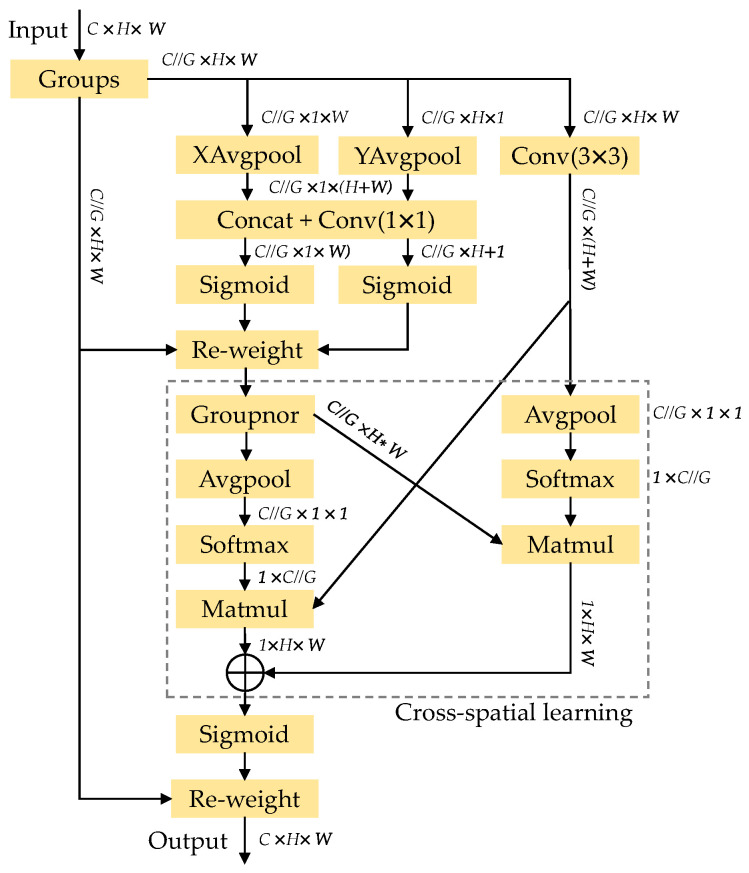
Efficient Multi-scale Attention Module.

The EMA structure, as shown in Figure 5, extracts the attention weights of the grouping feature maps through three parallel subnets, embedding precise positional information into EMA, and integrating contextual information of different scales to enable the convolution module to generate better pixel-level attention on advanced feature maps. Then, a cross-spatial learning method is used to enhance the network structure by handling short and long-term dependency relationships. In contrast to the progressive behavior formed by a limited receptive field, the parallel use of 3 × 3 and 1 × 1 convolutions allows for more contextual information to be utilized in the intermediate features, and finally, the fused features are refined to obtain the output result $f_{e}$.

**Figure 6:**
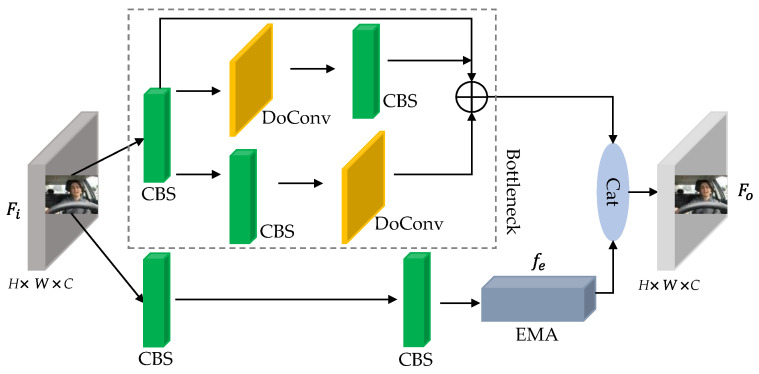
Lightweight Contextual-Information-Fusion Module.

The L-CIFM structure, as shown in Figure 6, consists of two convolutional branches and one residual edge for context feature extraction. Firstly, the upper-level feature $F_{i}$ is preprocessed with a 1 × 1 standard convolution and inputted into the bottleneck module for extracting adjacent contextual features. The left branch uses a 3 × 3 DoConv to enhance facial feature perception of the input feature, improving the deep network’s perception of global information on the original input image. Then, a 1 × 1 standard convolution is used to refine the rich semantic information from the upper level. The right branch first uses a 1 × 1 standard convolution to obtain the position information of facial labels and then enriches local features through a 3 × 3 DoConv. The contextual fusion feature is obtained by adding (⊕), and on this basis, the contextual fusion feature is concatenated with the upper-level information enhanced by EMA through Cat, realizing the merging of information from multiple feature dimensions and forming the lightweight contextual-information-fusion module. Finally, the expression for the output of the fused context feature information $F_{o}$ is:

(1) $$F_{o}=f^{1\times 1}\left(f^{1\times 1}f_{e}+f^{1\times 1}\left(f_{DOC}^{3\times 3}f^{1\times 1}⊕f^{1\times 1}f_{DOC}^{3\times 3}⊕1\right)\right),$$

In the equation, $f^{1\times 1}$ represents a 1 × 1 Conv, $f_{DOC}^{3\times 3}$ represents a 3 × 3 DoConv, and $f_{e}$ represents enhancing the attention on effective facial features of the upper layer input using EMA.

#### 2.2.4. Improvement of Loss Function

The loss function of bounding box regression mainly consists of three parts: bounding-box-localization loss, object-confidence loss, and object-classification loss. It is a key component of object detection and has a significant impact on the predictive performance of the model. The CIoU loss used in the baseline model does not effectively reflect the differences between the true values of width and height and their confidence. There is also an issue of imbalance in the bounding-box regression loss between high-quality and low-quality samples. This paper introduces the various losses for bounding-box regression proposed in reference [32], called WIoU. WIoU can be divided into WIoU v1 based on attention and WIoU v2 with a monotonically focusing mechanism (FM), as well as WIoU v3 with a dynamic non-monotonic FM. WIoU v3 can assign a smaller gradient gain to anchor boxes with larger outliers, effectively preventing large gradient loss in low-quality samples. This fully exploits the non-monotonic tuning potential of static FM, reduces the penalty for distance and aspect ratio on low-quality samples, speeds up the convergence of the model, and improves the extraction performance of facial local features. Therefore, this paper adopts WIoU v3 to reconstruct the loss function of the baseline model, effectively balancing the impact of high and low-quality sample differences on the model and improving the extraction performance of facial local features.

**Figure 7:**
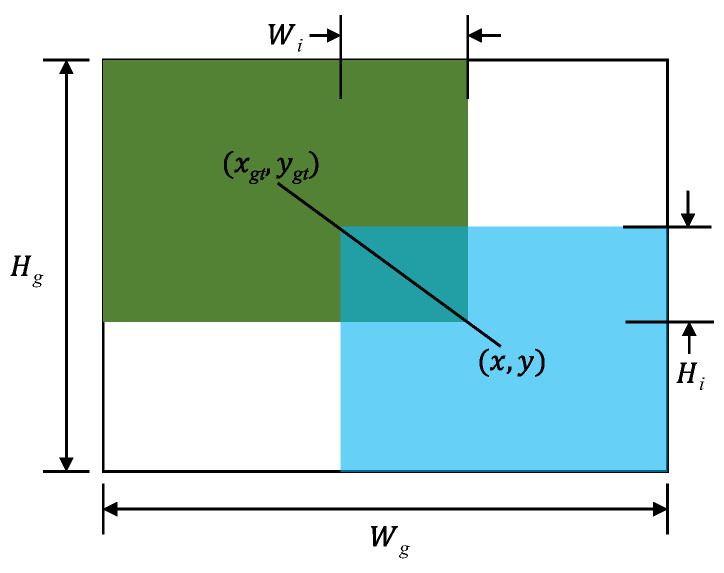
Illustration of Intersection over Union.

Taking Figure 7 as an example, the Intersection over Union (IoU) loss between the ground truth box (green) and the predicted box (blue) can be obtained, denoted as $L_{IoU}$:

(2) $$L_{IoU}=1-\frac{W_{i}H_{i}}{wh+w_{gt}h_{gt}-W_{i}H_{i}},$$

Then define the penalty factor $R_{WIoU}$ to amplify the $L_{IoU}$ of low-quality anchor boxes, and on this basis, adjust the focus point of bounding-box regression on the quality of anchor boxes using gradient gain r, thus obtaining the loss $L_{WIoUv3}$ of WIoU v3:

(3) $$R_{WIoU}=exp\left(\frac{{\left(x-x_{gt}\right)}^{2}+{\left(y-y_{gt}\right)}^{2}}{{W_{g}^{2}+H_{g}^{2}}^{*}}\right),$$

(4) $$r=\frac{\beta }{\delta \alpha^{\beta -\delta }},$$

(5) $$L_{WIoUv3}=rR_{WIoU}L_{IoU},$$

In the equation, outlier degree $\beta =\frac{L_{IoU}^{*}}{L_{IoU}}$; hyperparameters δ and α are used to control the mapping relationship between outlier degree β and gradient gain r.

**Figure 8:**
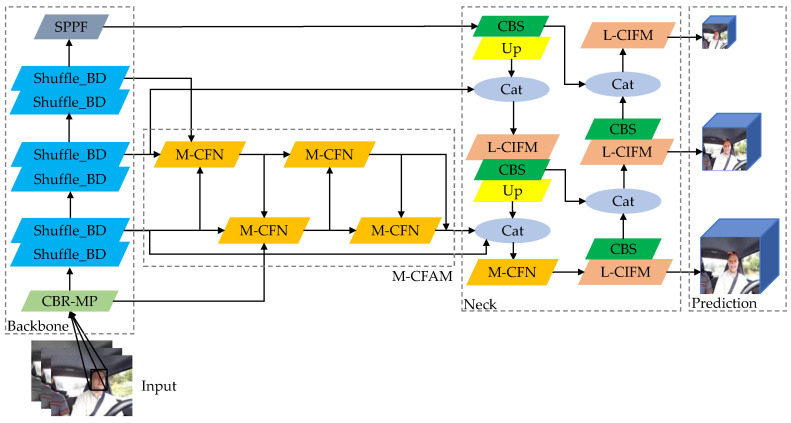
Improved Face-Detection Structure.

The improved face-detection model is shown in Figure 8.

## 3. Keypoints Extraction and Fatigue-Judgment Model Construction

### 3.1. Extraction of 3D Facial Keypoints

The localization coordinates of facial landmarks are crucial for calculating the aspect ratio of the eyes and mouth. Considering that the traditional MTCNN five-point localization only includes the positions of the left and right eyes, nose, and corners of the mouth, it can only locate the facial contour but cannot determine whether the person is in a fatigued state. Additionally, due to the adoption of a three-level cascaded network, the detection speed is slow. Moreover, the 2D 68 keypoints extracted by Dlib have the issues of losing feature information and poor real-time performance when the driver’s head rotates significantly. Therefore, in order to accurately, quickly, and stably extract facial keypoints and enhance the focus on semantically meaningful regions, this paper adopts a lightweight architecture called Attention Mesh [33] for predicting the coordinates of 468 facial landmarks, which directly predicts the positions of the vertices of a 3D facial mesh.

**Figure 9:**
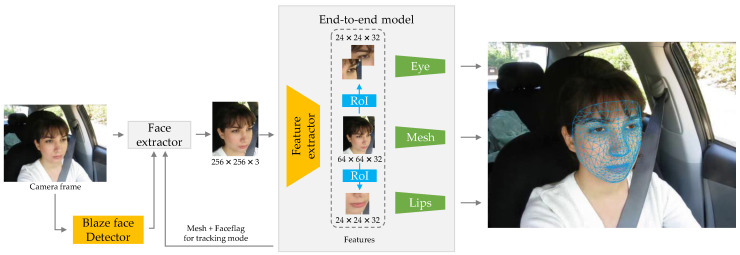
Structure of Attention Mesh model.

As shown in Figure 9, the implementation mechanism of 3D facial keypoints extraction consists of two parts: the face extractor and the end-to-end feature-extraction model. The input of the detection video frame image is provided either through the previous frame tracking or directly by the detector. Then, these inputs are divided into separate sub-models by the feature-extraction model, which directly extracts the predicted coordinates of the eye and mouth regions. Each sub-model can independently control the grid size of each feature region based on feature changes, thus improving the quality of grid coverage. Finally, a set of normalization is applied to horizontally align and evenly size the eye and mouth features, further improving the accuracy of prediction. Therefore, it can achieve the same or even higher accuracy in facial keypoint localization as multi-stage cascaded methods, while also improving the speed of localization extraction.

**Figure 10:**
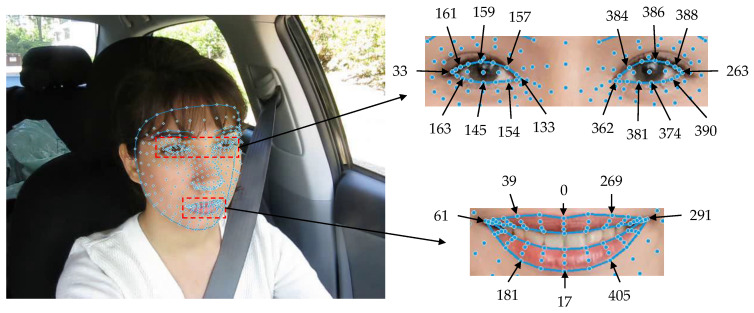
Facial 468 keypoints.

The visualization of 3D facial keypoints extraction is shown in Figure 10. The distribution of keypoints for the left and right eyes are 33, 133, 145, 154, 157, 159, 161, 163, 263, 362, 374, 381, 384, 386, 388, and 390. The distribution of keypoints for the outer contour of the mouth is 0, 17, 39, 61, 269, 181, 291, and 405.

### 3.2. Fatigued-Driving Detection Model with Feature Fusion

#### 3.2.1. Eye-Mouth Aspect Ratio and the Determination of Its Threshold

The height of the eye opening varies with blinking, fluctuating as it rapidly decreases and gradually approaches zero during the closing process. When opening, it maintains balance within a certain threshold range. Therefore, this paper proposes to assess the driver’s eye opening and closing situation by calculating the eye aspect ratio (EAR) as presented in reference [34] and obtaining the corresponding threshold value.

In addition to judging whether there is fatigued driving based on changes in the driver’s eyes, yawning is also a noticeable change in state. When a driver yawns, the distance between the upper and lower lips and the distance from the left corner of the mouth significantly increase and decrease, respectively, and they maintain a short period of stability within a certain threshold range. Therefore, in order to enrich the criteria for determining fatigued-related conditions, the mouth aspect ratio (MAR) is calculated based on the EAR, and the corresponding threshold is determined. Additionally, in order to prevent the problem of losing the keypoints of the inner contour due to different changes in mouth features among different drivers, this study extracts eight points from the external contour of the mouth to calculate the mouth aspect ratio.

The formulas for calculating EAR and MAR are as follows:

(6) $$EAR_{right}=\frac{∥Y_{384}-Y_{381}∥+∥Y_{386}-Y_{374}∥+∥Y_{388}-Y_{390}∥}{3∥X_{362}-X_{263}∥},$$

(7) $$EAR_{left}=\frac{∥Y_{161}-Y_{163}∥+∥Y_{159}-Y_{145}∥+∥Y_{157}-Y_{154}∥}{3∥X_{33}-X_{133}∥},$$

(8) $$EAR=\frac{EAR_{left}+EAR_{right}}{2},$$

(9) $$MAR=\frac{∥Y_{39}-Y_{181}∥+∥Y_{0}-Y_{17}∥+∥Y_{269}-Y_{405}∥}{3∥X_{61}-X_{291}∥},$$

In the equation, $X_{362}$, $X_{263}$, $X_{33}$, $X_{133}$ and $X_{61}$, $X_{291}$ represent the horizontal coordinates of four keypoints of the left and right eyes and two keypoints of the mouth outline, respectively. $Y_{384}$, $Y_{381}$, $Y_{386}$, $Y_{374}$, $Y_{388}$, $Y_{390}$, $Y_{161}$, $Y_{163}$, $Y_{159}$, $Y_{145}$, $Y_{157}$, $Y_{154}$, $Y_{39}$, $Y_{181}$, $Y_{0}$, $Y_{17}$, $Y_{169}$, and $Y_{405}$ represent the vertical coordinates of twelve keypoints of the left and right eyes and six keypoints of the mouth outline, respectively.

**Figure 11:**
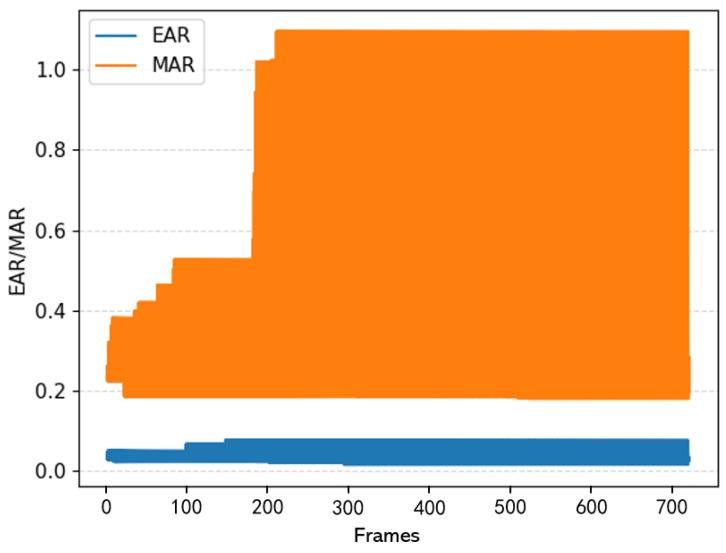
Analysis of EAR and MAR results.

As shown in Figure 11, a frame-by-frame analysis was conducted on the process of a driver transitioning from a normal state to a fatigued state of closing eyes and yawning, using randomly selected video data from the publicly available YawDD [35] simulated-driving dataset. From the graph, it can be observed that as the number of frames increases, the MAR remains between 0.2–0.3 when the driver is in a normal closed-mouth state. When yawning occurs, the MAR rapidly increases and stabilizes at around 1.1. Furthermore, the difference between yawning and regular mouth states is most obvious when the MAR exceeds 0.65. Therefore, 0.65 is determined as the threshold for yawning detection, meaning that when MAR > 0.65, the driver is considered to have yawned once. Similarly, based on the observation that the minimum EAR during eye closure remains around 0, a threshold of 0.02 is determined for eye-closure detection. Hence, when EAR < 0.02, the driver is considered to have closed their eyes once.

#### 3.2.2. The Number of Frames of Continuous Eye Closure and Yawning in a Single Instance

From Figure 11, it can be seen that during the process of fatigued driving, the number of continuous frames with closed eyes $F_{e}$ and continuous frames with yawning $F_{m}$ are significantly different from the normal driving state. Studies have shown that, under normal conditions, a person yawns for about 6.5 s [36], which is approximately 150 frames. Therefore, the determination of whether a driver is fatigued can be made by calculating the number of continuous closed-eye frames and continuous yawning frames. The calculation formula is as follows:

(10) $$F_{e}=F_{ej}-F_{ei},$$

(11) $$F_{m}=F_{mj}-F_{mi},$$

In the formula, $F_{ei}$, $F_{ej}$, $F_{mi}$, and $F_{mj}$, respectively, represent the starting and ending frames of closing eyes and yawning.

#### 3.2.3. PERCLOS Criteria and the Determination of Its Threshold

PERCLOS, which stands for Percentage of Eyelid Closure over the Pupil over Time, is a physical parameter used to determine driver fatigue. Taking the P80 measurement standard as an example, when the eye-blink ratio is below 0.2, it is considered as complete eye closure; when the ratio is above 0.8, it is considered as complete eye opening. If this value exceeds a certain threshold, it can be determined that the driver is in a fatigued state. Therefore, in order to make a more accurate determination of driver fatigue, the Percentage of Yawning in a Unit of Time is proposed. Within a specified unit cycle frame $F_{0}$, the PERCLOS scores for the eyes ($P_{eyes}$) and mouth ($P_{mouth}$) are calculated based on the total number of frames with eye closure and yawning.

(12) $$P_{eyes}=\frac{\sum_{F_{start}}^{F_{end}}\left(F_{ej}-F_{ei}\right)}{F_{0}},$$

(13) $$P_{mouth}=\frac{\sum_{F_{start}}^{F_{end}}\left(F_{mj}-F_{mi}\right)}{F_{0}},$$

In the equation, $F_{start}$ and $F_{end}$ represent the starting frame and ending frame of a specified unit cycle frame.

In order to determine the number of frames for continuous eye closure and continuous yawning, as well as the fatigue thresholds for the eyes and mouth, a detection experiment was conducted on the collected video dataset based on EAR and MAR thresholds. The total number of frames for eye closure and yawning within a unit cycle was counted, and the PERCLOS score was calculated. Based on the three calculation indicators mentioned above, a model for determining fatigued-driving detection was established. After analyzing the experimental results, it was determined that within a unit cycle of 150 frames ($F_{0}$ = 150 frames), if the PERCLOS score for the driver’s eyes and mouth is not less than 0.15, or the number of frames for continuous eye closure is not less than 20 frames, or the number of frames for continuous yawning is not less than 30 frames, then it is determined that the driver is in a state of fatigued driving. Otherwise, the driver is considered to be in a normal driving state. The fatigue-determination formula is as follows:

(14) $$\left\{\begin{array}{c} F_{0}=150\\ P_{eyes}=P_{mouth}\geq 0.15\\ F_{e}\geq 20\\ F_{m}\geq 30 \end{array}\right.,$$

**Figure 12:**
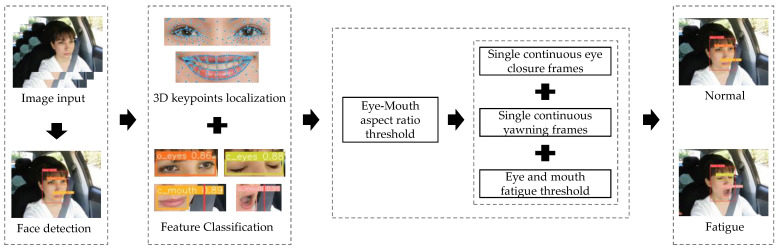
Fatigued-driving detection process.

The fatigued-driving detection process is shown in Figure 12. In order to address the problem of low detection accuracy caused by single-feature recognition or single-target detection based on eye and mouth in current methods, as well as the issue of serious false positives, false negatives, and keypoint loss caused by large head-pose variations of drivers, this paper combines the extraction of three-dimensional keypoints, eye-mouth aspect ratio calculation, and feature-classification detection to comprehensively determine the changes in driver’s blinking and yawning states. Specifically, when the driver’s mouth is open (o_mouth) and the mouth ratio exceeds the specified threshold, both conditions are met, and it is determined that the driver is yawning. When the driver’s eyes are closed (c_eyes) or the eye ratio is below the specified threshold, one of the conditions is met, and it is determined that the driver is blinking. The formulas for discriminating blinking and yawning are as follows:

(15) $$\left\{\begin{array}{c} o\_mouth\wedge \left(MAR>0.65\right)\\ c\_eyes\vee \left(EAR<0.02\right) \end{array}\right.,$$

Finally, a fatigued-driving detection model is built by integrating eye and mouth features. The current state of the driver, whether fatigued or not, is determined based on the number of continuous closed-eye frames, continuous yawning frames, and the fatigue thresholds for eyes and mouth. The model outputs two judgment results: normal driving and fatigued driving.

## 4. Analysis of Experimental Results

### 4.1. Dataset and Experimental Conditions

The dataset of this study consists of a total of 8021 images, including public datasets YawDD, CEW [37], DrivFace, and Drozy, which consist of male and female drivers of different races, with and without glasses, and in normal and fatigued driving states (speaking and non-speaking). Additionally, there are self-built video datasets of different drivers in real driving scenes during daytime and nighttime.

First, the video data of YawDD and self-built are mirrored, rotated, and cropped according to the time of capturing a picture every 20 frames. Then, the CEW, DrivFace, and Drozy databases are added to enrich the dataset and compensate for the lack of diversity and scene changes in YawDD and self-built video data, enhancing the robustness and generalization ability of the model. The dataset is annotated by using the Python annotation library, Labelimg, resulting in 8021 face bounding-box labels, 3579 o_mouth bounding-box labels, 4634 o_eyes bounding-box labels, 4310 c_mouth bounding-box labels, and 3166 c_eyes bounding-box labels, totaling 23,710 facial feature bounding-box labels across five categories. The dataset is then split into a training set and a validation set in an 8:2 ratio.

During model training, the number of epochs is set to 150. The image size is 640, and the batch size is 16. The training is conducted by using the rect matrix to reduce redundant padding in image preprocessing, decrease memory usage during training, and accelerate the inference process. The specific experimental conditions during training are shown in Table 1.

### 4.2. Face-Feature Detection Experimental Analysis

#### 4.2.1. Evaluation Indicators

This study evaluates the effectiveness of the improved face-detection algorithms by using the metrics of average precision (AP), mean average precision (mAP), floating-point operations (FLOPs), parameters (Params), and model size (Size). AP and mAP are used as performance evaluation metrics for target prediction. A higher value indicates a higher recognition rate for different categories of faces and a stronger overall performance of the model. The other metrics are used to evaluate the lightweight nature of the model. A smaller FLOPs value indicates lower computational complexity, while smaller Params and Size values indicate the model is lightweight. The formulas for calculating AP and mAP are as follows:

(16) $$P=\frac{TP}{TP+FP},$$

(17) $$R=\frac{TP}{TP+FN},$$

(18) $$AP=\int_{0}^{1}P(R)dR,$$

(19) $$mAP=\frac{\sum_{i=1}^{5}{AP}_{i}}{5},$$

In the formula, P and R represent precision and recall, respectively. TP represents the number of correctly predicted faces in each category by the model. FP represents the number of incorrectly predicted faces in each category by the model. FN represents the number of faces in each category that were not predicted by the model. AP represents the average precision of predicting each category in the face. The number 5 represents the 5 feature categories of faces being classified.

#### 4.2.2. Comparison of Main Branch Refactoring Experiment

Table 2 shows the experimental comparison results of introducing Shufflenetv2 and improved Shufflenetv2_BD for backbone reconstruction based on the baseline model. From the table, it can be seen that Shufflenetv2 achieves a relatively higher mAP of 92.7% with a minimal increase in parameters after the addition of DoConv, minimizing the overall performance loss of the model. Therefore, this paper chooses Shufflenetv2_BD to reconstruct the backbone of YOLOv5s, a lightweight network model.

#### 4.2.3. Ablation Experiment

In order to verify the effectiveness of the improved algorithm in facial-feature detection, ablative experiments are conducted to demonstrate the detection effects of various optimization points. Each group of experiments uses the same hyperparameters and training method, and the results are shown in Table 3.

It can be seen that compared with the baseline model, the improved algorithm achieves a significant improvement in how lightweight the model is, with a reduction of 56.3% in model size and 58% in parameters, and a floating-point operation of only 5.9 GFLOPs. After introducing the improved Shufflenetv2_BD, the AP50 of each category remains basically unchanged, but the mAP decreases by 2%, resulting in a significant loss. By aggregating shallow and deep texture features of faces across scales, enhancing the fusion of contextual features with semantic information, and redefining the loss function using WIoU, the AP50 and mAP are ultimately improved by 1.5% and 3%, respectively. Compared to the baseline model, there is an additional improvement of 1% and 1.7% for AP50 and mAP, demonstrating that the improved algorithm achieves stronger detection performance than the baseline model while being more lightweight than the reconstructed backbone.

**Figure 13:**
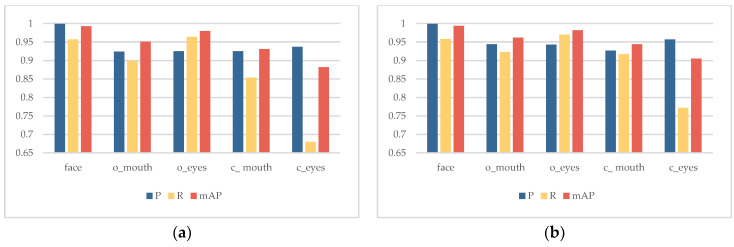
Classification Detection Results Before/After Improvement: (**a**) YOLOv5s; (**b**) Ours.

**Figure 14:**
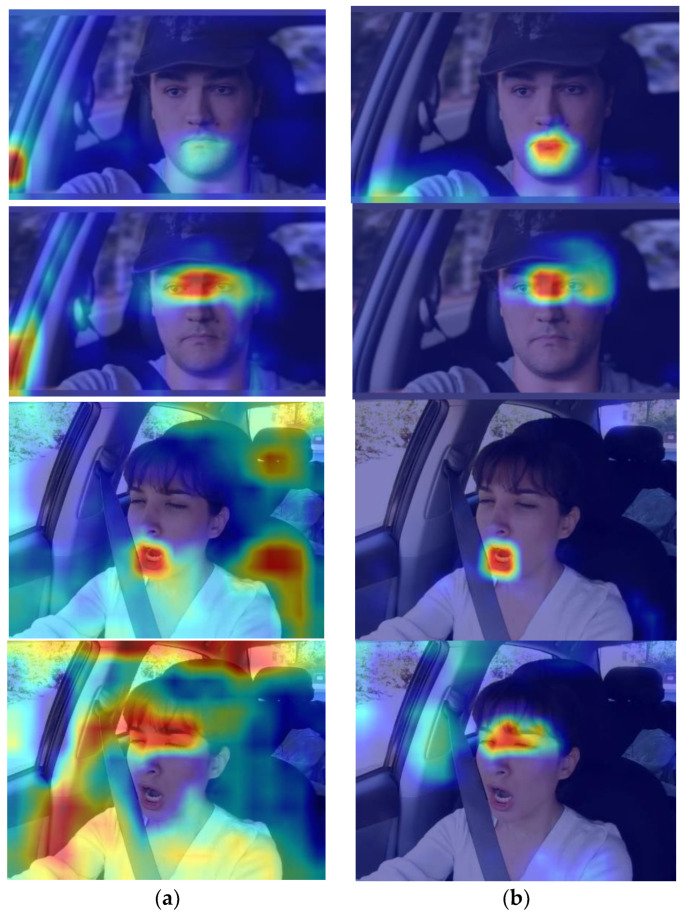
Comparison of heatmap results before and after improvement: (**a**) YOLOv5s; (**b**) Ours.

As shown in Figure 13 and Figure 14, the improved classification detection results and visualized heatmaps before and after improvement based on ablation experiments are used to demonstrate the advantages of the improved algorithm in predicting facial features of various categories. From the figures, it can be seen that the introduction of EMA can extract more complete facial-feature information, providing accurate location information for categories with smaller targets and shallower features, effectively enhancing the attention to feature regions, especially in the categories of c_mouth and c_eyes. The recall and mean average precision of c_eyes have the most significant improvement, reaching 9.2% and 2.3%, respectively.

The improved algorithm’s classification detection results were evaluated using 50,000 randomly selected face images with different feature variations and different scenes from the CelebA [38] face dataset. The corresponding classification recognition accuracy was used as the evaluation metric. As shown in Table 4, thanks to the fusion feature enhancement of M-CFAM and L-CIFM, the improved algorithm achieved a recognition rate of 98.6% for the target regions of facial features. Specifically, the recognition rates for the face and o_eyes categories reached almost 100%, while the c_eyes and c_mouth categories had relatively higher false detection rates and room for improvement in classification judgments. Nonetheless, the average recognition rate exceeded 97%, indicating a good evaluation performance.

The results show that although our method has a relatively smooth improvement in overall mAP, the recognition precision P of face, o_mouth, o_eyes, and c_mouth is also comparable to the baseline model. Given the point-by-point optimization improvement on the baseline model, it can achieve accurate extraction and prediction of various facial features and categories while ensuring extremely low system overhead, thereby verifying the effectiveness of the improved algorithm.

#### 4.2.4. Horizontal Network Comparison Experiment

Considering that this article mainly focuses on the lightweight real-time research and analysis of the fatigued-driving detection model, we have weighed the choice to conduct a comprehensive performance comparison with the current mainstream fatigued-driving detection algorithms. From Table 5, it can be seen that the model size of this algorithm is only 6.3 MB, much smaller than YOLOv3-Tiny, YOLOv4-Tiny, YOLOv7-Tiny, and SSD. It has a stronger advantage in lightweight terminal deployment. Even compared with YOLOv4-Tiny and YOLOv5n, which have similar lightweight levels, although the floating-point operation volume is slightly higher, this algorithm improves mAP by 7.6% and 2.9%, respectively, ensuring the overall performance of predicting facial-feature categories while making the model lightweight. In addition, compared with the most advanced object-detection algorithm, the YOLOv8 series, the algorithm proposed in this paper has a lower mAP by 2% and relatively weaker detection performance. However, it has a significant advantage in being lightweight and having a better overall performance. Therefore, it can be concluded that this algorithm can achieve high robustness with very low computational power, ensuring the portability requirement on embedded mobile terminals, further validating the advancement of this algorithm.

### 4.3. Fatigue Sample Test Result Analysis

The fatigued-driving detection model in this study integrates the weight file “best.pt” obtained during the training process of the improved algorithm into the judgment model in order to achieve the goal of multi-index and multi-feature fusion detection. Therefore, in order to verify the accuracy of the fatigued-driving detection model, 135 segments averaging 560 frames of untrained data were collected from the YawDD video dataset, including normal driving (speaking and not speaking) and fatigued driving videos of different driving scenarios, drivers wearing and not wearing glasses, and drivers of different genders, as instance samples. The recognition accuracy obtained from the instance detection is ultimately used as the evaluation metric, and the judgment results of the fatigued-driving detection model in this study are compared with the results in references [39,40], which adopt the MTCNN five-point positioning method, as shown in Table 6.

From Table 6, it can be seen that the fatigued-driving detection effect of reference [39] is actually poor, with weak discrimination ability for normal state, eye changes, and yawning. Among them, the fatigue recognition rate in the yawning state is the lowest, only 23.1%, and the overall performance is much lower than the 98.1% of reference [40] and the 96.7% of this study. Compared with reference [40], the fatigued-driving detection model constructed in this study, which combines eye and mouth features, achieves an overall judgment accuracy of 96.3%. The recognition accuracy of 45 fatigued driving samples and 90 normal driving samples reached 93.3% and 97.8%, respectively. Due to the consideration of eye fatigue, the overall comprehensive performance is slightly weaker than the 97% of reference [40]. However, if we ignore eye fatigue and keep the same conditions as reference [40], the overall accuracy of this study can reach 97.5%, which is the strongest performance. In addition, although the judgment methods and sample sizes used in reference [39] and reference [40] are different, they also reflect from the side that the fatigued-driving detection judgment model designed in this study has better fatigue-judgment results and a higher detection level. However, due to the lack of validation in real driving scenarios, the discrimination mechanism of the fatigued-driving detection model still has some mixed discrimination errors when the driver’s speaking state and fatigue features are not obvious. The classification discrimination ability of the model still needs further improvement. However, in overall detection and discrimination, our method has shown significant advantages when compared to other approaches.

**Figure 15:**
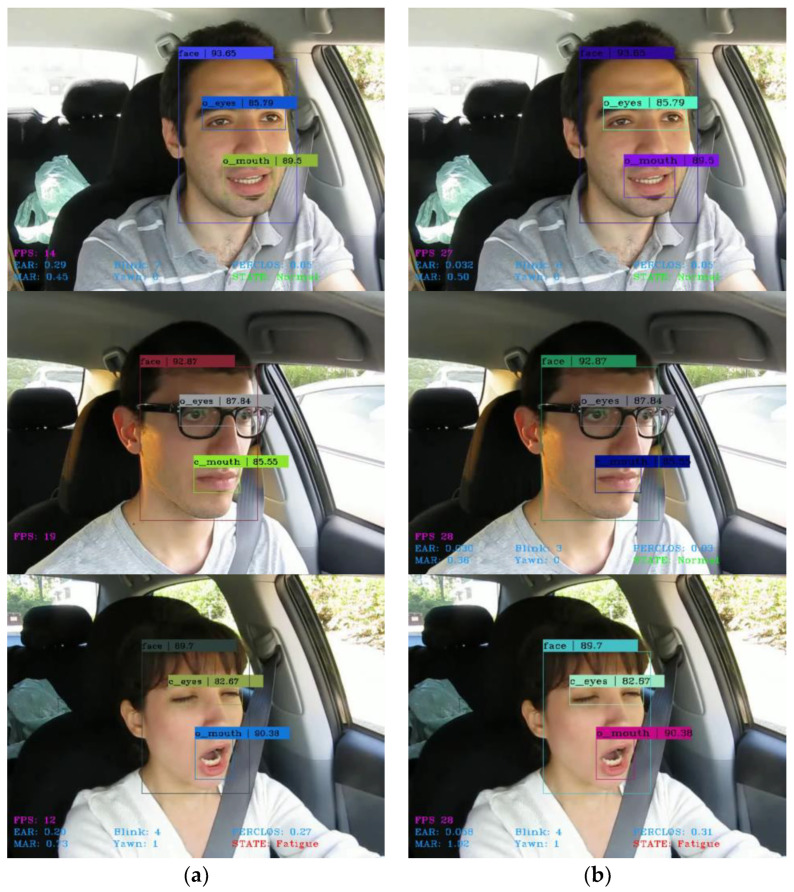
Detection results of instance samples based on different facial keypoint extraction methods: (**a**) Dlib; (**b**) Attention Mesh.

Figure 15 shows the comparison of detection results by adopting Dlib and Attention Mesh for keypoint extraction of normal driving and fatigued driving, wearing glasses, and not wearing glasses (with other variables kept the same). The EAR and MAR in the figure can dynamically detect and calculate the changes in the driver’s eyes and mouth in real time. When the driver closes their eyes or yawns, it indicates that the EAR or MAR is lower or higher than the set threshold, and “Blink” and “Yawn” will display the number of eye blinks and yawns. Then, the fatigue detection model will count the number of continuous eye closures and continuous yawns within a unit cycle frame, as well as the total number of eye closures and yawns, and display the calculated eye and mouth PERCLOS scores on the “PERCLOS”. If the score is lower than the specified threshold, it is determined as “Normal”, otherwise it is determined as “Fatigue”.

From the figure, it can be seen that the lightweight face detection algorithm designed in this study is able to accurately recognize various facial-feature categories. In addition, compared to Dlib, which has the problem of keypoint loss leading to the failure of the fatigue-detection model and poor real-time detection, the fatigue-detection model using Attention Mesh has a faster detection speed, reaching 28 FPS, which basically meets the requirements of real-time detection and verifies the effectiveness of Attention Mesh in quickly and stably extracting keypoints. The final result achieves a good continuous detection effect, which is in line with the experimental design.

## 5. Discussion and Conclusions

This paper introduces a lightweight and robust driver-face detection method and a fatigued-driving determination model. Firstly, Shufflenetv2_BD is used to reconstruct the backbone of YOLOv5s, achieving a lightweight network and an improved training speed of deep linear networks. Secondly, M-CFAM is introduced between the backbone and neck networks to enhance the cross-scale fusion of facial features and to reduce the loss of shallow features. Then, L-CIFM is introduced to enhance the extraction ability of facial-region features by the neck network. In addition, in order to accelerate the convergence speed of the model, WIoU is introduced as a new loss metric, and the loss function is redefined. Through comparative experiments, the proposed algorithm reduces the parameters and model size by 58% and 56.3% compared to the baseline model, and the floating-point operations are only 5.9 GFLOPs. The mAP on the self-built dataset reaches 95.7%, an improvement of 1%. This indicates that the algorithm not only performs well in terms of being lightweight but also effectively improves the detection performance. Finally, based on the threshold value of the eye-mouth aspect ratio calculated by three-dimensional keypoints and the detection of facial-feature categories, the designed fatigued-driving determination model comprehensively judged 135 instance samples within a specified unit cycle frame by using the number of frames of continuous eye closure and continuous yawning as well as the threshold of eye-mouth PERCLOS, achieving a recognition accuracy of 96.3%. A high level of fatigued-driving detection has been achieved. This achieves a recognition accuracy of 96.3% and reaches a high level of fatigued-driving detection. Thus, it verifies that the face-detection algorithm and fatigue-determination model can effectively detect and determine drivers in real-time in different driving scenarios, different genders, and different driving characteristics, demonstrating strong robustness and providing support for the transplantation and deployment of fatigued-driving detection.

The algorithm in this paper is designed for fatigued-driving detection, and it has achieved good results. However, it lacks consideration for extremely complex scenes and face-occlusion problems. In the future, we will increase the coverage of data in complex scenes and introduce tracking algorithms to optimize the insufficient or lost feature extraction in extreme driving scenarios with occlusions in order to improve the applicability in multiple scenarios.

## Figures and Tables

**Table 1:** Experimental conditions.

Experimental Environment	Details
CPU	Xeon (R) Platinum 8255C
GPU	RTX 2080Ti (11 GB)
Memory	40 GB
Deep learning framework	Pytorch 1.11.0
Programming language	Python 3.8
GPU acceleration tool	CUDA 11.3

**Table 2:** Validation Experiment of Improved Shufflenetv2_BD Backbone.

Method	mAP	FLOPs/G	Params	Size [MB]
YOLOv5s	0.947	15.8	7.02 M	14.4
Shufflenetv2	0.918	5.9	3.24 M	6.8
Shufflenetv2_BD	0.927	5.9	3.36 M	7.0

**Table 3:** Experimental Results of Ablation.

YOLOv5s+Shufflenetv2_BD	L-CAM	L-CIFM	WIoU	AP50	mAP	FLOPs/G	Params	Size [MB]
				0.938	0.947	15.8	7.02 M	14.4
**√**				0.940	0.927	5.9	3.36 M	7.0
**√**	√			0.958	0.953	7.5	3.44 M	7.2
**√**		√		0.943	0.948	4.3	2.74 M	5.9
**√**			√	0.946	0.932	5.9	3.36 M	7.0
**√**	√	√		0.953	0.950	5.9	2.95 M	6.3
**√**	√	√	√	0.955	0.957	5.9	2.95 M	6.3

**Table 4:** Evaluation Results of the Improved Algorithm’s Classification Detection.

Classes	Quantity	Correctly Identify the Number	Accuracy
face	50,000	49,850	0.997
o_mouth	22,383	22,025	0.984
o_eyes	28,850	28,590	0.991
c_mouth	26,899	26,280	0.977
c_eyes	19,517	18,834	0.965
Comprehensive	147,649	145,579	0.986

**Table 5:** Horizontal Comparative Validation Results of Mainstream Algorithms on Dataset.

Method	mAP	FLOPs/G	Params	Size [MB]
SDD	0.774	28.4	20.52 M	105.1
YOLOv3-Tiny	0.876	13.1	8.72 M	51.4
YOLOv4-Tiny	0.881	3.4	6.06 M	22.4
YOLOv5n	0.928	4.1	1.77 M	3.8
YOLOv7-Tiny	0.956	13.1	6.02 M	12.3
YOLOv8s	0.977	28.8	11.20 M	23.7
YOLOv5s	0.949	15.8	7.02 M	14.4
Ours	0.957	5.9	2.95 M	6.3

**Table 6:** Fatigue Detection Identification Results and Comparison.

Method	Category	Quantity	Correctly Identify the Number	Error Category	Accuracy
	Normal	45	45	-	1
	Speaking	45	43	Tired eyes + yawning	0.956
Ours	Eye fatigue	15	13	Normal	0.867
	Yawning fatigue	30	29	Normal	0.967
	Comprehensive	135	130	-	0.963
	Normal	110	67	Tired eyes + yawning	0.609
	Speaking	100	97	Tired eyes + yawning	0.970
Reference [39]	Eye fatigue	33	23	Normal	0.696
	Yawning fatigue	82	19	Normal	0.231
	Comprehensive	325	206	-	0.634
	Normal	10,291	9643	Tired yawning	0.937
	Speaking	12,904	12,614	Tired yawning	0.978
Reference [40]	Eye fatigue	-	-	-	-
	Yawning fatigue	21,643	21,234	Normal	0.981
	Comprehensive	44,838	43,491	-	0.970

## Data Availability

Data are available on request due to restrictions, e.g., privacy or ethical restrictions.

## References

[1] Amodio A., Ermidoro M., Maggi D., Formentin S., Savaresi S.M. (2018). Automatic detection of driver impairment based on pupillary light reflex. IEEE Trans. Intell. Transp..

[2] Sikander G., Anwar S. (2018). Driver fatigue detection systems: A review. IEEE Trans. Intell. Transp..

[3] Chai M. (2019). Drowsiness monitoring based on steering wheel status. Transp. Res. D Trans. Environ..

[4] Jeon Y., Kim B., Baek Y. (2021). Ensemble CNN to detect drowsy driving with in-vehicle sensor data. Sensors.

[5] Xi J., Wang S., Ding T., Tian J., Shao H., Miao X. (2021). Detection Model on Fatigue Driving Behaviors Based on the Operating Parameters of Freight Vehicles. Appl. Sci..

[6] Zhang G., Etemad A. (2021). Capsule attention for multimodal EEG-EOG representation learning with application to driver vigilance estimation. IEEE Trans. Neural System. Rehabil..

[7] Satti A.T., Kim J., Yi E., Cho H.Y., Cho S. (2021). Microneedle array electrode-based wearable EMG system for detection of driver drowsiness through steering wheel grip. Sensors.

[8] Qiu X., Tian F., Shi Q., Zhao Q., Hu B. Designing and application of wearable fatigue detection system based on multimodal physiological signals. Proceedings of the IEEE International Conference on Bioinformatics and Biomedicine (BIBM).

[9] Dinges D.F., Grace R. (1998). PERCLOS: A Valid Psychophysiological Measure of Alertness as Assessed by Psychomotor Vigilance.

[10] Dziuda Ł., Baran P., Zieliński P., Murawski K., Dziwosz M., Krej M., Piotrowski M., Stablewski R., Wojdas A., Strus W. (2021). Evaluation of a fatigue detector using eye closure-associated indicators acquired from truck drivers in a simulator study. Sensors.

[11] Alioua N., Amine A., Rziza M. (2014). Driver’s fatigue detection based on yawning extraction. Int. J. Veh. Technol..

[12] Zhang W., Su J. Driver yawning detection based on long short term memory networks. Proceedings of the IEEE Symposium Series on Computational Intelligence (SSCI).

[13] Knapik M., Cyganek B. (2019). Driver’s fatigue recognition based on yawn detection in thermal images. Neurocomputing.

[14] Zhang K., Zhang Z., Li Z., Qiao Y. (2016). Joint face detection and alignment using multitask cascaded convolutional networks. IEEE Signal. Proc. Let..

[15] King D.E. (2009). Dlib-ml: A machine learning toolkit. J. Mach. Learn. Res..

[16] Deng W., Zhan Z., Yu Y., Wang W. Fatigue Driving Detection Based on Multi Feature Fusion. Proceedings of the IEEE 4th International Conference on Image, Vision and Computing (ICIVC).

[17] Liu W., Tang M., Wang C., Zhang K., Wang Q., Xu X. Attention-guided Dual Enhancement Train Driver Fatigue Detection Based on MTCNN. Proceedings of the International Academic Exchange Conference on Science and Technology Innovation (IAECST).

[18] Liu Z., Peng Y., Hu W. (2020). Driver fatigue detection based on deeply-learned facial expression representation. J. Vis. Commun. Image R..

[19] Zhang N., Zhang H., Huang J. Driver fatigue state detection based on facial key points. Proceedings of the International Conference on Systems and Informatics (ICSAI).

[20] Li K., Gong Y., Ren Z. (2020). A fatigue driving detection algorithm based on facial multi-feature fusion. IEEE Access..

[21] Babu A., Nair S., Sreekumar K., Karuppusamy P., Perikos I., García Márquez F.P. (2022). Driver’s drowsiness detection system using Dlib HOG. Ubiquitous Intelligent Systems.

[22] Cai J., Liao X., Bai J., Luo Z., Li L., Bai J. (2023). Face Fatigue Feature Detection Based on Improved D-S Model in Complex Scenes. IEEE Access..

[23] Lin T.Y., Dollár P., Girshick R., He K., Hariharan B., Belongie S. Feature pyramid networks for object detection. Proceedings of the IEEE Conference on Computer Vision and Pattern Recognition (CVPR).

[24] Liu S., Qi L., Qin H., Shi J., Jia J. Path aggregation network for instance segmentation. Proceedings of the IEEE/CVF Conference on Computer Vision and Pattern Recognition.

[25] Zheng Z., Wang P., Ren D., Liu W., Ye R., Hu Q., Zuo W. (2021). Enhancing geometric factors in model learning and inference for object detection and instance segmentation. IEEE Trans. Cybern..

[26] Ma N., Zhang X., Zheng H.T., Sun J. (2018). Shufflenet v2: Practical guidelines for efficient cnn architecture design. Proceedings of the Computer Vision-ECCV 2018: 15th European Conference.

[27] Zhang X., Zhou X., Lin M., Sun J. Shufflenet: An extremely efficient convolutional neural network for mobile devices. Proceedings of the IEEE/CVF Conference on Computer Vision and Pattern Recognition (CVPR).

[28] Cao J., Li Y., Sun M., Chen Y., Lischinski D., Cohen-Or D., Chen B., Tu C. (2022). Do-conv: Depthwise over-parameterized convolutional layer. IEEE Trans. Image Process..

[29] Guo X. (2023). A novel Multi to Single Module for small object detection. arXiv.

[30] Liu S., Huang D., Wang Y. (2018). Receptive field block net for accurate and fast object detection. Proceedings of the Computer Vision-ECCV 2018: 15th European Conference.

[31] Ouyang D., He S., Zhang G., Luo M., Guo H., Zhan J., Huang Z. Efficient Multi-Scale Attention Module with Cross-Spatial Learning. Proceedings of the IEEE International Conference on Acoustics, Speech and Signal Processing (ICASSP).

[32] Tong Z., Chen Y., Xu Z., Yu R. (2023). Wise-IoU: Bounding Box Regression Loss with Dynamic Focusing Mechanism. arXiv.

[33] Grishchenko I., Ablavatski A., Kartynnik Y., Raveendran K., Grundmann M. (2020). Attention mesh: High-fidelity face mesh prediction in real-time. arXiv.

[34] Soukupova T., Cech J. Real-time eye blink detection using facial landmarks. Proceedings of the 21st Computer Vision Winter Workshop.

[35] Abtahi S., Omidyeganeh M., Shirmohammadi S., Hariri B. YawDD: A yawning detection dataset. Proceedings of the 5th ACM Multimedia Systems Conference.

[36] Gallup A.C., Church A.M., Pelegrino A.J. (2016). Yawn duration predicts brain weight and cortical neuron number in mammals. Biol. Lett..

[37] Weng C.H., Lai Y.H., Lai S.H. (2017). Driver drowsiness detection via a hierarchical temporal deep belief network. Proceedings of the Computer Vision-ACCV 2016 Workshops: ACCV 2016 International Workshops.

[38] Liu Z., Luo P., Wang X., Tang X. Deep learning face attributes in the wild. Proceedings of the IEEE International Conference on Computer Vision (ICCV).

[39] Ji Y., Wang S., Zhao Y., Wei J., Lu Y. (2019). Fatigue state detection based on multi-index fusion and state recognition network. IEEE Access..

[40] Liu W., Qian J., Yao Z., Jiao X., Pan J. (2019). Convolutional Two-Stream Network Using Multi-Facial Feature Fusion for Driver Fatigue Detection. Future Internet.

